# The Unification of the Public Health Services

**Published:** 1918-02-16

**Authors:** 


### RECONSTRUCTION AND THE POOR LAW.

### The Unification of the Public Health Services.

"The unique opportunity of the passing into law of the National Insurance Act, the grant of old-age pensions, the enlargements of the scope and authority of the County Councils and their medical officers, the increasing importance in the national economy of the great public infirmaries and asylums—all have come without that necessary union into one co-ordinated scheme of public assistance which should, and which ultimately must, do away with the anomalies, the overlapping, and the waste of our multiple petty 'authorities.' . . . Foremost amongst changes in administration must come Poor-Law reform."

(The Hospital, September 2, 1916.)

Nearly a decade has passed since the prolonged and painstaking inquiries of the Poor-Law Commission ended. Although the Majority and Minority Reports agreed in so far as to advocate a practical abolition of the Poor-Law system nothing has been done to put the recommendations of the Commission into force; Poor-Law reform has been shelved and legislation has passed it by. Now we are in the fourth year of a war of nations such as the world has never before known, and such as it could not have been believed to be capable of sustaining; this might have been expected to act as a deterrent to altruistic schemes of local government, and it would be strange indeed were the social upheavals which it has brought in its train to bring about that reorganisation which has for so long, in time of peace, proved a stumbling-block to politicians. Yet such an eventuality is by no means impossible. The Local Government Committee of the Ministry of Reconstruction, which was appointed in July last to "consider and report upon the steps to be taken to secure the better co-ordination of Public Assistance in England and Wales," has reported in favour of the transference, root and branch, into other hands of the services at present entrusted to Poor-Law authorities and the consequent elimination of the Poor-Law Guardian, the workhouse, the casual ward, and (in name at least) the pauper and the relieving officer. This is recommended as the first necessary step in the unification of the Public Health Services.

The Committee considers that the urgent need for a better organisation and the prevention of overlapping in public assistance "is not a matter of Poor Law only," but that the largest and most intractable case of overlapping is that of the Boards of Guardians on the one hand, and the county, municipal, and other health and education authorities on the other. "For the last decade Parliament has been unwilling to entrust the Boards of Guardians with new functions." "The resulting confusion has been aggravated by the growing popular prejudice against the Poor Law—a prejudice which does less than justice to the devoted work of the Guardians, and the continuous improvement in Poor-Law administration, especially in respect of the children and the sick."

It is in attempts to deal with children and, especially, with the sick that the Poor-Law system has attained its greatest successes, and yet made manifest its failure. The system was essentially unfitted to assume throughout the country that control of great State hospitals which has been thrown upon it. Popular satisfaction with—perhaps we should rather say popular tolerance of—the Poor-Law organisation began to wane with the demand for better conditions and better treatment for the sick "pauper" and the "pauper-child." Fifty years ago the Poor-Law hospitals or infirmaries began to be evolved upon the basis of the workhouse sick-wards. Slowly and laboriously these institutions, separated from workhouse control but never fully from workhouse associations, have improved; the more enlightened Boards of Guardians setting an example in their management, the less enlightened having the force of example thrust upon them. These State hospitals have become, in many places, worthy of the name: in buildings, equipment, dietary, and nursing they have progressed with the advance of the medical sciences, as have the voluntarily supported general hospitals; but, whilst comparable with the latter, they have always lagged behind them and have been held inferior in popular estimation. Nevertheless, with the improved treatment thus made available, the public distaste for going into "the House" has, in a considerable degree, ceased to extend to its separate infirmary or hospital section, and the anomalous position has arisen that this has come to be largely occupied by persons who are not destitute, who would not have sought "relief" but for their sickness, and of whom many pay for the treatment afforded them. Meanwhile the aged and infirm, for whom these institutions were originally intended, are often relegated to sick wards in the workhouses, which, excellently administered though they now are, are none the less "workhouse wards." Under the present system of control, moreover, there exist the widest variations (amounting to utter confusion) as to the degree of destitution which constitutes eligibility for admission; character or severity of illness and urgency of need for treatment are only too often not taken into account. The space at our disposal permits only of the mention of the important work done in Poor-Law institutions under the provisions of the Lunacy Acts and in connection with maternity, and of the vast specialised organisation in London of the Metropolitan Asylums Board, a body in some degree emancipated from baneful Poor-Law influences. The point which we wish to emphasise is that by far the greater proportion of hospital beds throughout the country is under the control and management of the Poor-Law authorities.

The Guardians of the poor should never have become, and should not be allowed to remain, governors of the large rate-supported hospitals. These Boards of Guardians are fettered to, and tied by, a cumbrous, antiquated, and effete system, characterised by a policy of "deterrence" which finds its expression in the application of "the

**RECONSTRUCTION AND THE POOR LAW**—(*cont.*)

workhouse test" and a rigorous and often harsh delimitation of out-relief. It is in London and other great towns that the shortcomings of the system and of the Guardians have become most obvious. The outlook of the Guardians is narrow, and they tend to become "guardians of the rates rather than guardians of the poor." They have frequently proved unsympathetic, even antagonistic, with regard to relief in its medical aspect, and any success in the development of the institutions for the sick under their management has resulted mainly from the efforts of the medical profession and the inspectors of the Local Government Board, aided by the influence of the press and the force of public opinion. Poor-law Guardians have shown a persistent jealousy of their medical officers and a constant fear that they have not over them the control which they deem to be necessary. The medical staff of their institutions has been always utterly inadequate and hopelessly over-burdened. The Guardians have acquiesced in nurse-training rather because it offered a cheap means of getting work done than from any real interest in its benefits or advantages. The popular prejudice against the Poor Law and everything appertaining to it is fully recognised in the service: tentative efforts have been made to counteract it, such as the substitution of the term "poor person" for "pauper." Workhouses, too, have often of late been termed "institutions," a mild euphemism at which certain authorities of the 'nineties would have stood aghast!

The proposal of the Committee, by whom it is desired to secure "the widest publicity and the fullest discussion," is to merge all the functions of the Poor-Law authorities in those of the County Councils and the County Borough Councils, and to transfer to them all Poor-Law buildings, to be by them appropriated and adapted as may be found most convenient. Provision for all children able to attend school should be made by the Local Education Committee. Further recommendations concern provision by the Councils for the unemployed able-bodied and for persons needing home assistance, the latter including old-age pensioners.

The changes proposed will undoubtedly meet with determined opposition from the Boards of Guardians and, at first, from some sections of those employed in the Poor-Law Services. The Committee, however, advises that the County Council Committees should be augmented, in undertaking some of their new duties, by the addition of persons possessing experience of various kinds in the work to be done, and, in the first instance, by some who have been members of Poor-Law authorities. This suggestion, which is intended to assist administration during a difficult period, offers an opportunity to those Guardians whose interest in their work is keen and unselfish to continue it in new companionship, and is therefore calculated to lessen the force and destroy the unanimity of the opposition which Boards of Guardians are sure to offer to disruption. It is recognised that the Poor-Law officers (amongst whom are included many experienced administrators, highly trained in investigation, registration, and the distribution and application of relief in many forms) are at present rendering valuable services to the community and are entitled to be treated with every consideration. It is recommended that they be transferred, subject to the option of the councils and of themselves, under schemes to be approved by the Local Government Board; that suitable provision be made for compensation when necessary; and that rights of tenure of office and of superannuation be fully protected.

We trust that the report and its recommendations will receive the publicity and discussion which the Committee desires. The changes foreshadowed are of the greatest importance and interest, embracing as they do the assignment of the control and management in the future of the great State hospitals of England and Wales.

